# Driving factors of urban community epidemic prevention and control capability: QCA analysis based on typical cases of 20 anti-epidemic communities in China

**DOI:** 10.3389/fpubh.2023.1296269

**Published:** 2024-01-05

**Authors:** Ruyi Shi, Bo Lu, Yiwen Zhong

**Affiliations:** ^1^School of Public Policy and Management, China University of Mining and Technology, Xuzhou, China; ^2^School of Safety Engineering, China University of Mining and Technology, Xuzhou, China; ^3^China North Industries Corporation, Beijing, China

**Keywords:** community resilience, COVID-19 epidemic, community epidemic prevention and control capacity, qualitative comparative analysis, China

## Abstract

**Introduction:**

In the wake of the COVID-19 outbreak, urban communities have emerged as the frontline defenders in epidemic prevention and control, providing the most effective means of curbing the spread of virus both inward and outward. This study attempts to explain the underlying factors and mechanisms that shape the community epidemic prevention and control capacity (CEPCC).

**Methods:**

We adopted a resilience-based perspective and drew on a sample of 20 exemplary anti-epidemic communities in China. By constructing an analytical framework and employing the fuzzy set qualitative comparative analysis method (fsQCA), we explored how four conditional variables—infrastructure completeness, community self-organizing ability, redundancy of community resources, and stability of regional economic development—and their various configurations impact the CEPCC.

**Results:**

Our findings reveal that the four conditional variables, when considered in isolation, cannot effectively enhance the CEPCC. Instead, four configuration pathways with mixed conditional variables exist. Notably, community self-organizing ability emerges as a vital condition for effectively strengthening the CEPCC.

**Discussion:**

This study identifies four pathways to improve the CEPCC and confirms the validity of the data results through case studies. Conclusions of this research contribute to a more nuanced understanding of the factors influencing the CEPCC, which can help communities to better plan and prepare for future epidemics and ensure better response and adaptation to the impacts of future emergencies.

## 1 Introduction

In the current era, the global environment and society are undergoing rapid changes, and the complexity of cities is constantly increasing. Urban public safety is facing severe challenges, such as high vulnerability and increasing uncertainty of risks, and various “black swan” or “gray rhinoceros” incidents are becoming more frequent. Enhancing the resilience governance capacity of cities to cope with various uncertain risks has become an important goal in the construction of resilient cities in the new era. The community is the basic unit of human collective activities and an important part of grassroots social governance in cities (1). The uncertainty of a risky society places greater governance pressures on communities and requires community governance to demonstrate stronger systemic adaptability and more sustainable governance effectiveness. In recent years, with the gradual shift of the focus of social governance to the grassroots level, communities have become increasingly important sites for resisting sudden incidents under a risk society. They are key entities in preventing and responding to various emergencies and play an important role in the entire emergency response system throughout all phases.

Since the outbreak of the COVID-19 epidemic, communities have become the frontline defenders in epidemic prevention and control, and a key line of defense in joint prevention and control. The COVID-19 epidemic has tested the community epidemic prevention and control capacity (CEPCC), as well as their effective governance and resilience. In the fight against the COVID-19 epidemic, communities served as nodes to build a close-knit anti-epidemic network through the interconnection of urban community nodes, playing an important role in preventing and resolving epidemic risks. However, by looking back at the epidemic prevention and control practices in various places, the performance of different urban communities in resolving and resisting epidemic risks and quickly restoring and stabilizing community functions is uneven. This exposed some problems in the current stage of community resilience construction in the face of the pressure of epidemic prevention and control and rapid recovery in China. In terms of organizational systems, the mechanism of community epidemic prevention and control is not perfect, the contingency plans are insufficient, there are loopholes, and the prevention and control capacity is inadequate (2). In terms of collaborative governance, the connection between departments is not smooth, the responsibility relationship at grassroots level is unclear, and there is obvious segmentation (3). In terms of community resource construction, there is a lack of emergency professional and technical personnel, insufficient emergency resource reserves, and blockages in the connection with social resources (4).

The notion of community resilience, as a proactive response to disaster threats, has garnered persistent attention in recent years (5). Community resilience provides a reliable anchor to guarantee continued community governance functions and facilitates a seamless transition between “smooth governance” under normal circumstances and “emergency governance” in crisis situations. Community resilience building can effectively activate community emergency response capabilities, reduce community vulnerability, and is also an important way to enhance community anti-epidemic capabilities and optimize the national public health emergency prevention and control system (6). Against this backdrop of research background and contemporary concerns, this paper explores the complex linkages between the factors that drive the CEPCC from the perspective of community resilience. The study seeks to address the following questions: What are the primary determinants that impact the CEPCC? What are the configuration paths to enhance the CEPCC? Based on 20 exemplary anti-epidemic community cases in China, multi-case analysis is conducted to investigate the specific characteristics and grouping patterns of the variables that affect the CEPCC. This paper aims to provide a thorough understanding of the complex mechanism of driving factors that affect the CEPCC and enable effective responses to public health emergencies in community scale.

## 2 Literature review and research framework

### 2.1 Literature review

The concept of resilience in ecology was first introduced by ecologist Holling in 1973, where he used the term to describe the multi-steady-state feature of ecosystems, becoming the origin of resilience theory (7). With the three paradigm shifts of engineering resilience, ecological resilience, and evolutionary resilience, the concept and definition of resilience theory have been widely applied and developed in various fields such as psychology, economics, engineering, biology, etc. Along with the advancement of industrialization and urbanization and the display of urban vulnerability, urban and community resilience have gradually become hot topics among scholars. Community resilience is a more precise interpretation of the resilience city concept in spatial dimensions (4). Many studies have defined community resilience, with most of the definitions emphasizing the buffering, bearing, and recovery capacities of communities when facing disasters or risk impacts. However, there is no unified definition of community resilience, with most definitions focusing on the completeness of resources, abilities, and internal conditions of communities. Peacock et al. defined community resilience from the perspective of capital, stating that community resilience should include social capital, economic capital, material capital, human capital, and other resources (8). Bruneau et al. believed that community resilience is the ability of a community to reduce the likelihood of, absorb, and recover from impacts caused by disaster (9). Coles and Buckle considered community resilience to be the speed and ability of a community to recover from the impacts and pressures of a disaster to its original state (10). In this paper, community resilience is defined as the ability of a community to mitigate and resolve crises in the face of sudden events using its own community resources and protection capacities, guaranteeing the normal functioning of the community's original functions and quickly recovering from the crisis.

The concept of epidemic prevention and control emerged in response to the COVID-19 virus and has not yet been clearly defined by the academic community. According to the classification standard for emergency events, epidemics belong to public health emergencies. Therefore, relevant concepts of public health and disease control can be used to define the CEPCC. Epidemic prevention and control refer to the scientific organization and implementation of prevention and control measures and strategies for various diseases, ensuring their effective prevention and control, and preventing their occurrence, development, and spread. Wang and Zhao summarized the disease prevention and control capacities into disease prevention capacities, disease control capacities, and emergency response capacities for public health emergencies from the perspective of the response process of public health emergencies (11). In terms of the importance of the work, the core of disease prevention and control is to protect patients, staff, residents, and anyone entering health facilities from communicable infections (12). Based on the definitions of disease prevention and control capability and community governance capability, and in conjunction with the unique attributes of epidemic and other public health emergencies, the CEPCC can be summarized as follows: the CEPCC refer to the comprehensive ability of communities to prevent and deal with the development and spread of epidemics and to reduce human casualties and economic losses by mobilizing and integrating resources such as people, finances, materials, and information in responding to epidemic outbreaks or diffusion. The CEPCC are the driving force generated by the community subject in response to public health emergencies such as epidemics, and it is the comprehensive capability to prevent and control the spread of epidemics and safeguard the life, health, and safety of residents.

Communities play a pivotal role in epidemic control, as their ability and effectiveness in this regard can directly impact the spread of viruses and social stability (13). Epidemic prevention and control, as a form of crisis management, tests the governance system and capacity of communities. Strengthening the construction of community governance systems, transforming governance resources into governance capacity, and enhancing self-service capacities are vital for effective pandemic prevention and control. The CEPCC is closely intertwined with their resilience in the face of crises. Resilient communities have demonstrated stronger resistance and recover quicker than traditional communities amidst the impact of epidemics (14). Since the outbreak of COVID-19, numerous scholars have researched the factors that impact the CEPCC. Galbusera et al. identified infrastructure as a critical factor that influences the ability of urban communities to combat epidemics, including elements such as basic necessities, protective equipment, public health resources, and emotional resources (15). Furthermore, Cui et al. conducted comprehensive research on the CEPCC and concluded that economic level, social networks, emergency response and daily management, medical resources and public services, as well as experiences in disasters and information technology, are key factors that influence this capacity, in addition to the availability of hardware and software resources such as infrastructure and management capacity (14). On the other hand, some scholars have highlighted the role of the government in influencing the CEPCC. Chu et al. emphasized the importance of urban governance capacity in epidemic prevention and control, including government guidance and support, central government support for labor, financial subsidies and material resources, among others (16). In addition, factors such as a prolonged state of emergency, vulnerability of health care systems, inadequate infrastructure and resources, poor technological and management capacities hinder prevention and control efforts and exacerbate the spread of epidemics (17, 18).

In summary, existing research has revealed multiple contributing factors to the CEPCC, providing insights for better understanding this capacity. However, there are still limitations that need to be addressed. Firstly, current analyses lack a systematic theoretical framework for examining factors that impact the CEPCC. Secondly, most research focuses solely on identifying influencing factors, with little exploration of their interrelationships. Based on this, this paper aims to introduce resilience theory and build an analytical framework to explore the factors that affect the CEPCC. In addition, most current research on epidemic prevention and control relies on text analysis, content analysis, and case analysis, with few conducting comparative analyses of multiple cases. This paper conducts multi-case studies to explore the complex relationships and configuration paths between the driving factors of the CEPCC.

### 2.2 Research framework

Existing research on community resilience has mainly focused on theoretical models of urban community resilience, community resilience assessment, and community resilience building. In terms of community resilience building, Bruneau et al. proposed that community resilience must have four attributes: robustness, redundancy, resourcefulness, and rapidity (9). Norris et al. proposed the NCRM community resilience model, which includes four aspects: economic development, community capacity, information and communication, and social capital, from the perspective of disaster risk reduction (19). Miles proposed the WISC conceptual model, and considered community, infrastructure, ecosystems, socio-economic, and community capital as important content for resilience building (20). Regarding community resilience assessment, a relatively mature evaluation framework has been formed, as shown in Table 1, which searches for key elements to enhance community resilience through multidimensional evaluation. The most classical examples include the CDRI (21), the DROP (22), and the PEOPLES multi-dimensional community resilience assessment indicator (23).

**Table 1:** Division of community resilience dimensions by some scholars.

**References**	**Division of community resilience dimensions**
Cutter et al. (22)	Ecological resilience, social resilience, economic resilience, institutional resilience, infrastructure resilience, and community capacity
Bruneau et al. (9)	Technological resilience, organizational resilience, social resilience, and economic resilience
Renschler et al. (23)	Population structure, ecological environment, government services, physical infrastructure, lifestyle and community capacity, economic development, and cultural (social) capital
Joerin et al. (24)	Physical resilience, social resilience, economic resilience, institutional resilience, and natural environmental resilience
Wilson (25)	Social capital, economic capital, and environmental capital
Alshehri et al. (26)	Health and wellbeing, governance, physical facilities and environment, economy, information and communication, and society
Qasim et al. (27)	Social resilience, economic resilience, institutional resilience, and physical resilience
Chong et al. (28)	Economy, society/culture, and environment/physics/infrastructure/system
Peacock et al. (8)	Social, economic, physical, human, and natural capital
Almutairi et al. (29)	Governance and institutions, infrastructure, environment and climate change, and social economy

**Figure 1:**
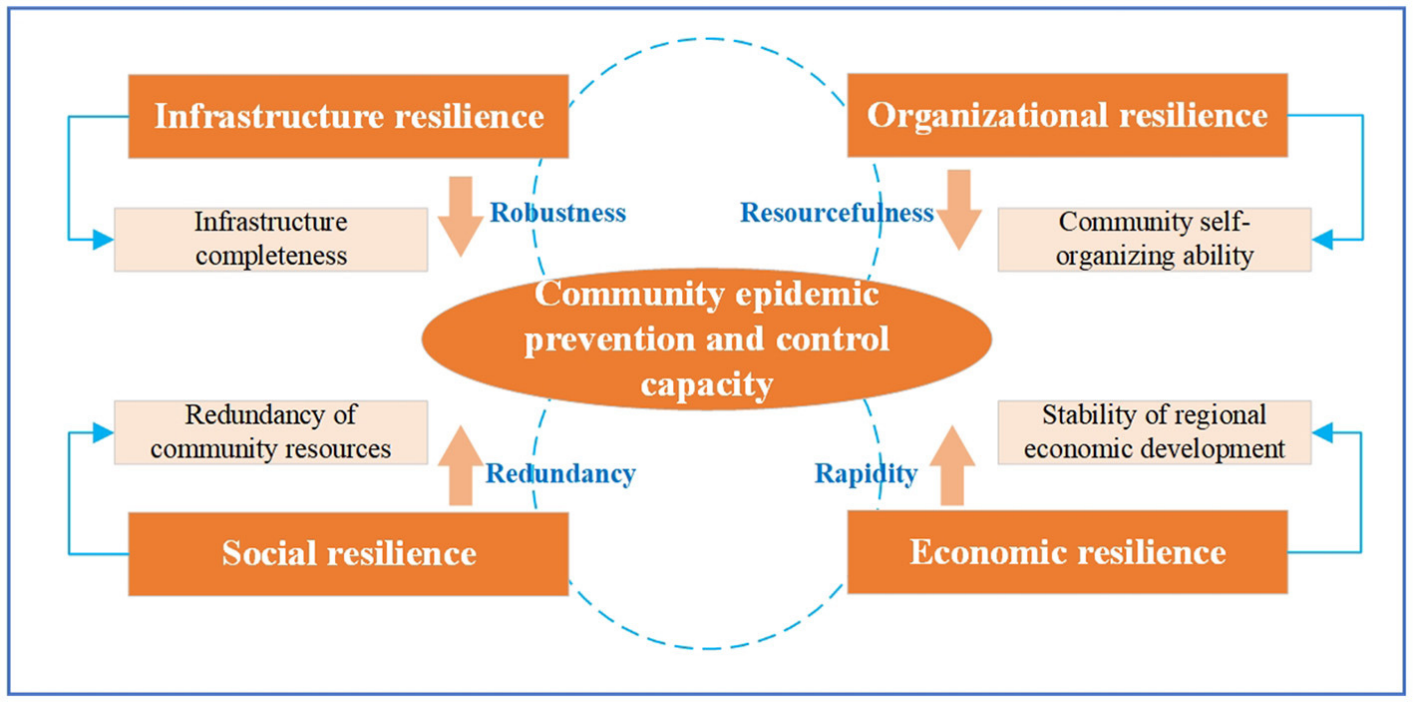
Research framework.

Academia deconstructed the concept of resilience from different dimensions, but most of the existing research on community resilience is based on the context of natural disasters, which cannot effectively provide guidance for enhancing community resilience in the context of public health emergencies. Communities represent the “final outpost” of urban governance, and their capacity for epidemic prevention and control is a critical component of modern community governance rooted in the provision of public safety services. It is not only an essential aspect of emergency response capacities but also a reflection of governance effectiveness. The use of community epidemic prevention and control systems to achieve “dynamic clearing of COVID-19 cases” and respond to sporadic outbreaks has helped to mitigate the impact and damage of epidemics on society, and this feat owes much to the support provided by community resilience (30). The community resilience theory provides a new perspective for recognizing and improving the CEPCC. Integrating community resilience into the community governance structure can enhance the stability of the community governance system and the community's capacity for adaptability and rapid recovery in response to social changes. Drawing on the aforementioned discussion, this study combined community resilience theory with the CEPCC. Based on the characteristics of CEPCC and public health emergencies, this study integrated and improved the resilience frameworks put forward by Bruneau et al. and Cutter et al. in Table 1, and developed a CEPCC analytical framework. A dynamic analysis is carried out on various factors and path configurations that promote the advancement of the CEPCC (as shown in Figure 1). The analytical framework comprises four dimensions: infrastructure resilience, organizational resilience, social resilience, and economic resilience. In the context of community epidemic prevention and control, infrastructure resilience aims at minimizing the damage inflicted on the community system and its infrastructure during an epidemic outbreak while ensuring that the community system retains all its functions. Organizational resilience refers to the community's organizational capacity to respond promptly, make scientifically informed decisions, and execute them effectively during an epidemic. Social resilience is demonstrated by the community's ability to recover independently, relying on the redundancy of community resources to replace any lost function. Economic resilience plays a crucial role in determining the availability and quantity of resources that a community has during and after an epidemic shock. This impact affects the rate and effectiveness of recovery and reconstruction efforts during an epidemic.

## 3 Comparative analysis of factors affecting the CEPCC

### 3.1 Methodology

**Figure 2:**
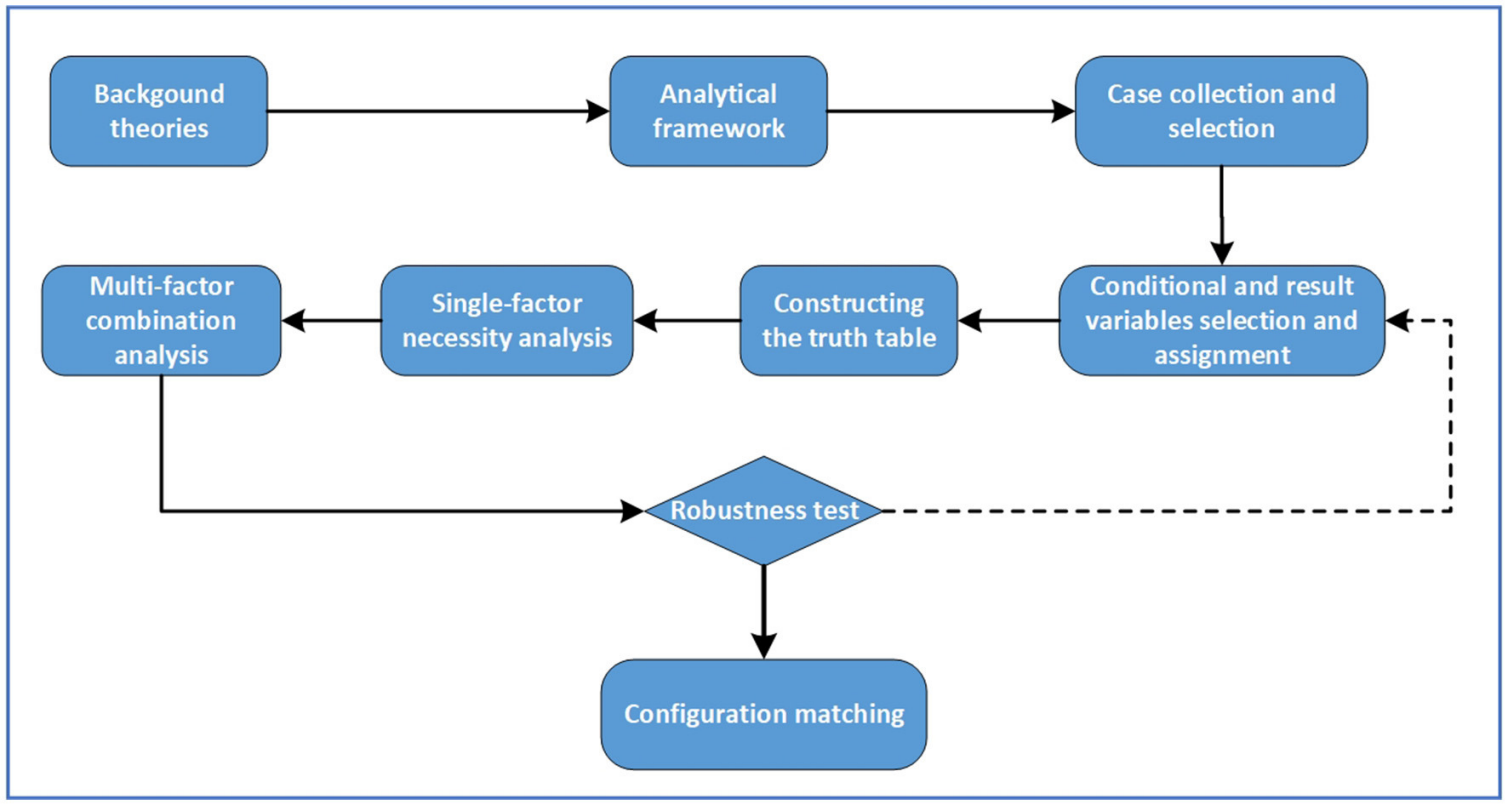
Process of the fsQCA method.

Qualitative comparative analysis (QCA) is a research method based on set theory and Boolean algebra developed by American sociologist Ragin in the 1980's to study causal complexity in real society (31). The QCA method combines the advantages of case analysis and quantitative analysis, and focuses on the complex causal relationships between sets of conditions and outcomes from a holistic perspective. The QCA is categorized into three types: clear set QCA (csQCA), multivalued QCA (mvQCA), and fuzzy set QCA (fsQCA). Considering that the CEPCC is the result of various factors through multiple combinations of pathways, it is difficult to systematically and comprehensively explore the complex interaction between multiple influencing factors based on the traditional binary relationship of “independent variable—dependent variable.” In this paper, fsQCA is used to study the factors influencing the CEPCC. Compared with csQCA and mvQCA, fsQCA has the advantages of both qualitative and quantitative analysis, which can deal with the partial membership problem between sets, explain the degree of change in the values of variables, and help to analyze the causal complexity between the antecedent conditions and the outcome variables at a deeper level. These features make fsQCA well-applicable to the analysis of factors influencing the CEPCC. This paper analyzes the influencing factors of the CEPCC based on 20 typical cases of anti-epidemic community, considers each case as a grouping of conditional variables, and comparatively discusses different cases to find out the asymmetric multiple concurrent causality. Accordingly, the key factors affecting the CEPCC and their grouping paths can be derived. The process of the fsQCA method is depicted in Figure 2.

### 3.2 Case selection

The sample case library used in this paper is derived from the “National Urban Community Epidemic Prevention and Control Excellent Case Collection Activity” launched by the Chinese Society of Social Governance in 2020. A total of 359 cases were initially screened from 25 provinces, municipalities, and autonomous regions, including Beijing. These 359 urban community cases were identified as the primary case library for this study to ensure the representativeness and scientific nature of the sample cases. Three principles were followed in the selection of cases to ensure the representativeness and comparability of the sample cases: Firstly, the principle of diversity. The selected communities in this study reflect the diversity of geographic distribution and economic conditions as much as possible. Secondly, the principle of comparability. According to the requirements of the outcome variables set in this study, the presentation of case samples ensures both the sufficient homogeneity of the overall case library and the maximum heterogeneity within the case library (32). Thirdly, the principle of data availability. QCA requires sufficient data to explain the cases, so the selected cases need to have detailed case information that is readily available and verifiable.

According to information from different sources such as the official website of the Chinese Society of Social Governance, local government statistics, and authoritative media reports, the primary case library was further screened to determine the final typical case library. Finally, the information of the typical sample cases was collected and organized, and the information from different sources was logically verified and screened to produce 20 sample cases. This study combined case data with variable assignment rules to form a truth table and input it into the fsQCA software for calculation. The sample case table is shown in Table 2.

**Table 2:** Samples of epidemic prevention cases in urban communities.

**S/N**	**Province/city**	**Title of case**
1	Jinfeng Town, Chongqing High-tech Zone	“Party Building + Epidemic Prevention:” Building a “Five-Color Group Yang” Governance Pattern in Jinfeng Town, Chongqing High-tech Zone
2	Xincheng Community, Xianshuigu Town, Tianjin	“Three-Community Cooperation” + “Four-Party Coordination:” Smart Community Governance in Epidemic Prevention and Control
3	Huojiaying Community, Changping District, Beijing	Only Under Adversity Can We See the True Colors of Grass, and Only under Fire Can We See True Gold: Huojiaying Community's Fine Management Model Revealed in Epidemic Prevention and Control
4	Ranyi New Town, Gaogeng Subdistrict, Qionglai City, Chengdu, Sichuan Province	How to Build Community Governance Community for Rural Residents in Concentrated Living Areas in Epidemic Prevention and Control: A Case Study of Ranyi New Town, Gaogeng Subdistrict, Qionglai City
5	Buerjin County Committee Political and Legal Committee of the Communist Party of China, Xinjiang	Building a Four-Level Joint Defense Model of “Party Organization + Comprehensive Governance Center + Grid + Double-Linked Households” to Strengthen the “Community Defense Line” in Epidemic Prevention and Control
6	Fuxing Community, Jinbi Subdistrict, Xishan District, Kunming City, Yunnan Province	“Five Hearts” Co-construction of the Fuxing Community to Win the Epidemic Prevention and Control War
7	Ruquan Community, Duting Subdistrict Office, Lichuan City, Enshi State, Hubei Province	“436” Epidemic Prevention Work Method of Ruquan Community
8	Xiaoshan District, Hangzhou City, Zhejiang Province	Xiaoshan City Brain Xiaoshan Platform “Xiaoshan's Battle Against Epidemic-Epidemic Comprehensive Management System”
9	Xingfu Township People's Government, Nanguan District, Changchun City, Jilin Province	Creating a Three-Dimensional Work System for Urban Epidemic Prevention and Control
10	Huaihe Subdistrict, Erqi District, Zhengzhou City	“Five Hearts and One Network” to Win the Battle against COVID-19
11	Hailar District, Hulunbuir City, Inner Mongolia Autonomous Region	“654” Work Method to Aid Epidemic Prevention and Control
12	Haiyu Community Party Committee, Xin'an Subdistrict, Baoan District, Shenzhen City, Guangdong Province	Haiyu Community's “Party-Mass Pioneer Station” in Small Residential Communities
13	Nanhu Community, Yunhe District, Cangzhou City, Hebei Province	Party Building Leads the Southlake Community to Play the Most Beautiful Anti-Epidemic Symphony
14	Gulou District Civil Affairs Bureau, Fuzhou City, Fujian Province	Gulou Community “Happiness Pass”
15	Lihua Community, Beida Subdistrict, Liangxi District, Wuxi City, Jiangsu Province	“Whole New Epidemic Prevention” Overcomes Difficulties, Creating a High-quality Answer Sheet for Lihua Community
16	Fengshun Community, Longwangtang Subdistrict, Gaoxin District, Dalian City, Liaoning Province	Epidemic Prevention and Control Electronic Passes of Fengshun Community, Longwangtang Subdistrict
17	Jianzhu Subdistrict Office, Xiangfang District, Harbin City, Heilongjiang Province	“Party Building Leading and Social Cooperation:” Solving the Problem of Abandoned Communities in Epidemic Prevention and Control
18	Haijiao Subdistrict, Haicheng District, Beihai City, Guangxi Province	Establishing the “Three-Dynamic” Mechanism to Reinforce the Front-line Defense Network in Hitting the Epidemic
19	Xinzhou Convergence Media Service Center, Shanxi Province	“Xinzhou Click-to-Shoot Platform” under the Propaganda Department of the CPC Xinzhou City Committee
20	Jinputao Community, Dawei Town, Baohe District, Hefei City, Anhui Province	Writing a New Chapter in The City's United Fight Against the Epidemic.

### 3.3 Variable selection and assignment

Based on the community resilience theory analysis framework, a research model containing four conditional variables and one outcome variable was formed and corresponding variables were set. In this paper, we use the four-valued fuzzy set assignment method in the fsQCA to assign values to the data, set the variables based on the degree of affiliation to a fixed value between 0 and 1 (33).

Conditional variable 1: Infrastructure completeness, denoted as V1. Infrastructure is a key element of community resilience and directly affects the community's response capacities in emergency situations (34). Infrastructure, as the physical foundation of the community and the vehicle for the fulfillment of the community's means of resistance to the epidemic, plays a key role in the dynamics of urban disease transmission and control, and is an important cornerstone of the community's healthy living environment (35). In the context of the COVID-19 epidemic, this study sets the conditional variable under the infrastructure resilience dimension as infrastructure completeness, which specifically refers to the completeness of the community's epidemic prevention and inspection facilities, community healthcare facilities, and living services and security facilities in the process of fighting the epidemic. By improving community infrastructure, especially public health infrastructure, a key line of defense can be constructed to block the spread of the epidemic, thereby protecting the lives and health of residents (36). For example, community health-care organizations and staff take active interventions through differential diagnosis, isolation monitoring and medication control of infected persons. These initiatives mitigate the health damage of infected persons and protect vulnerable groups from infection. In this paper, we set the assignment rules of condition variable 1 based on the content of multiple cases and extensive literature analysis as follows: whether the epidemic prevention project is sound, such as emergency isolation sites, epidemic prevention inspection stations, safety signs, etc.; whether the medical and healthcare facilities and medical professional teams are sufficient; and whether the notification of epidemic information and the publicity of epidemic prevention knowledge are timely and comprehensive; whether the supply and protection facilities for basic necessities of life are complete, with a score of 1 when all four criteria were met; a score of 0.67 when three criteria were met; a score of 0.33 when two criteria were met; and a score of 0 when one or fewer criteria were met.

Conditional variable 2: Community self-organizing ability, denoted as V2. Community self-organizations serve as liaisons between the government and community residents, bridging the short-term failures of government functions during the epidemic (37). Community self-organizing ability mainly refers to the current ability of communities to organize activities, manage, innovate, expand, and sustain development. In this study, it refers to the leadership, organization, and management abilities of urban government and community organizations in the prevention and control of the COVID-19 epidemic. This conditional variable corresponds to the organizational dimension in the community resilience theory. In the process of community epidemic prevention and control, communities need to scientifically formulate prevention and control plans, allocate resources reasonably, and coordinate joint efforts by volunteers and residents in various departments to fight against the epidemic. The community's self-organizing ability is an important factor in measuring the improvement of the CEPCC. This study selected the establishment of an emergency leadership group against the epidemic as the standard rule for measuring community self-organizing ability. If the emergency leadership group against the epidemic was established, a score of 1 was assigned, and vice versa a score of 0 was assigned.

Conditional variable 3: Redundancy of community resources capacity, denoted as V3. This conditional variable corresponds to the social dimension in the community resilience theory. The abundance and effective utilization of community resources have a significant impact on the emergency response capacities of communities (38). The redundancy of community resources can provide necessary support for communities in emergency situations and ensure the normal functioning of basic community functions. It refers to the human, financial, policy and material resources invested in the process of community resistance to the epidemic. Human resources refer to the personnel available for the CEPCC, including community staff and volunteers. Epidemic prevention funds are allocated for the purchase of community anti-epidemic materials, disinfection and sterilization of public areas, and dissemination of anti-epidemic knowledge. Community anti-epidemic policies encompass policies and documents promulgated by the local government to support community epidemic prevention and control. Material reserves comprise of emergency epidemic prevention materials, medical supplies, and essential livelihood materials. This study selected the adequacy of epidemic prevention and control funding, policy support, community volunteers, and emergency supplies as the criteria for measuring redundancy of community resources capacity. When the case met all four criteria, a score of 1 was assigned; when three criteria were met, a score of 0.67 was assigned; when two criteria were met, a score of 0.33 was assigned; and when one or fewer criteria were met, a score of 0 was assigned.

Conditional variable 4: Stability of regional economic development capacity, denoted as V4. It describes the size, speed, and level of economic development in a certain region. Regions with higher and more stable economic development levels have stronger abilities to utilize and allocate resources. This conditional variable corresponds to the economic dimension in the resilience community theory. Developed regions, compared to less developed regions, have richer resource reserves and stronger resource allocation abilities, which have an important impact on supporting communities to recover from disaster states and maintaining community economic stability. In this study, the standard rules for measuring the stability of regional economic development capacity were borrowed from Zhang et al. (39). The local city where the urban community is located was measured based on whether it is a first-tier city, a second-tier city, whether the local GDP has reached the average level, and whether the local GDP has not reached the average level. A score of 1 was assigned when criterion 1 was met; a score of 0.67 was assigned when criterion 2 was met; a score of 0.33 was assigned when criterion 3 was met; and a score of 0 was assigned when criterion 4 was met.

Outcome variable: Enhancement of community epidemic prevention and control capacity. Since the cases for this study were selected from the evaluation of “National Urban Community Epidemic Prevention and Control Excellent Cases,” the assigning rules for the enhancement of CEPCC were set based on whether the case received other awards, was shortlisted as an excellent epidemic prevention case, was shortlisted in the evaluation of community epidemic prevention cases, or none of the aforementioned four standards were met. The judgment criteria for the “received other awards” standard was based on whether the case, corresponding local department, or community received commendations from subdistrict-level or higher units. Assigning a score of 1 when a single case met criteria 1, 2, and 3; a score of 0.67 when criteria 2 and 3 were met; a score of 0.33 when only criterion 3 was met; and a score of 0 when criterion 4 was met. The conditional variables, outcome variable, and assigning rules constructed in this study are presented in Table 3.

**Table 3:** Variable description and assignment rules.

**Categories**		**Name**	**Assignment rules**	**Assignment**
Outcome variable	Prevention and control results	Enhancement of community epidemic prevention and control capacity	1. Whether or not other awards have been received. 2. Whether or not shortlisted as an excellent epidemic prevention case. 3. Whether or not shortlisted in the evaluation of community epidemic prevention cases. 4. None of the aforementioned standards were met.	1. Score of 1 assigned when criteria 1, 2, and 3 are met. 2. Score of 0.67 assigned when criteria 2 and 3 are met. 3. Score of 0.33 assigned when only criterion 3 is met. 4. Score of 0 assigned when criterion 4 is met.
Conditional variables	Infrastructure resilience	Infrastructure completeness	1. The soundness of epidemic prevention engineering facilities (emergency isolation sites, epidemic prevention inspection stations, safety signs, etc.). 2. Medical and healthcare facilities and medical professional teams. 3. Epidemic information notification and prevention knowledge promotion. 4. Facilities for securing the supply of basic necessities.	1. Score of 1 assigned when all four criteria are met. 2. Score of 0.67 assigned when three criteria are met. 3. Score of 0.33 assigned when two criteria are met. 4. Score of 0 assigned when one or fewer criteria are met.
	Organizational resilience	Community self-organizing ability	1. The establishment of emergency leadership groups for epidemic prevention and control. 2. The failure to establish emergency leadership groups for epidemic prevention and control.	1. Score of 1 assigned when criterion 1 is met. 2. Score of 0 assigned when criterion 2 is met.
	Social resilience	Redundancy of community resources	1. Sufficient epidemic prevention funds. 2. Policy support availability. 3. Adequate number of community volunteers. 4. Adequate emergency supplies.	1. Score of 1 assigned when all four criteria are met. 2. Score of 0.67 assigned when three criteria are met. 3. Score of 0.33 assigned when two criteria are met. 4. Score of 0 assigned when one or fewer criteria are met.
	Economic resilience	Stability of regional economic development	1. Whether it is a first-tier city or not. 2. Whether it is a second-tier city or not. 3. Whether the local GDP has reached the average level. 4. Local GDP is not up to par.	1. Score of 1 assigned when criterion 1 is met. 2. Score of 0.67 assigned when criterion 2 is met. 3. Score of 0.33 assigned when criterion 3 is met. 4. Score of 0 assigned when criterion 4 is met.

### 3.4 Results and discussion

#### 3.4.1 Analysis of necessary conditions for the CEPCC

Before analyzing the conditional grouping, it is necessary to test whether each condition constitutes a necessary condition for the result. In the necessity testing of individual conditions, the criteria include consistency and coverage. Consistency is used to determine whether a particular condition variable is a sufficient or necessary condition for the outcome variable (40). The calculation formula is: *Consistency* **(X**_**i**_**≤Y**_**i**_**)=**∑**[***min* **(X**_**i**_**, Y**_**i**_ )]/∑**X**_**i**_ , where ***X*** represents the conditional variables, and ***Y*** represents the outcome variable. When the consistency is >0.8, it indicates that the conditional variable is a sufficient condition for the outcome variable. When the consistency is >0.9, it indicates that the conditional variable is a necessary condition for the outcome variable. After determining the sufficient or necessary conditions, the coverage rate can be used to further determine the explanatory power of the condition variable *X* on the outcome variable *Y* (40). The calculation formula is: *Coverage* (**X**_**i**_**≤Y**_**i**_)**=**∑**[***min* **(X**_**i**_**, Y**_**i**_ )]/∑**Y**_**i**_ , where the larger the value of the coverage indicator is, the greater the explanatory power of the condition variable on the outcome variable is. By using fsQCA 3.0 software to analyze each condition variable, the test results are shown in Table 4. According to Table 4, the consistency results of the four conditional variables in the hypothesis are all >0.8. Among them, the consistency result for the infrastructure completeness capacity is 0.971698 and that for the community self-organizing ability is 0.943396, both of which are >0.9, indicating that these two conditions are necessary conditions, i.e., core conditions, for the outcome variable. This indicates that there is a 97% probability that the infrastructure completeness capacity will emerge and a 94% probability that the community self-organization capacity will emerge in the process of community epidemic prevention and control. It has been proved that in China's past battles of epidemic prevention and control, the influence of both the capacity of complete infrastructure setup and the capacity of community self-organization on the community's ability to prevent and control epidemics has been crucial. Meanwhile, Table 4 shows that the consistency result for the redundancy of community resources is 0.857633, and that for the stability of regional economic development is 0.829331, both of which are >0.8, suggesting that these two conditions are sufficient conditions for the outcome variable, but further investigation is required in conjunction with the configuration results of other conditional variables. In cases where a single conditional variable is unable to meet the necessary condition criteria outlined in the consistency judgment standard, i.e., when the consistency is below 0.9, configuration analysis is necessary. By conducting configuration analysis, the impact of different combinations of each conditional variable on the outcome variable can be determined.

**Table 4:** Results of single-conditional variable analysis.

**Conditional variables**	**Consistency**	**Coverage**
V1	0.971698	0.707241
~V1	0.283019	0.829146
V2	0.943396	0.611111
~V2	0.056604	0.330000
V3	0.857633	0.730994
~V3	0.368782	0.680380
V4	0.829331	0.724345
~V4	0.368782	0.646617

#### 3.4.2 Analysis of conditional configurations for the CEPCC

Before conducting the conditional combination analysis, it is essential to establish the appropriate frequency and consistency thresholds to ensure the reliability of the research findings. This paper utilizes a sample size of twenty, which falls within the small to medium sample range. Referring to the research of Schneider and Wagemann (33), this study sets the frequency threshold to 1 and the consistency threshold to 0.8. By using the fsQCA 3.0 software, a standard analysis was conducted resulting in three different outcomes: complex solution, intermediate solution, and parsimonious solution, as depicted in Table 5. The complex solution does not consider logical residuals and excludes all counterfactual combinations, which is relatively complex. The parsimonious solution covers all logical residuals and is relatively simple but tends to ignore meaningful variables. However, the intermediate solution combines the strengths of the other two solutions and considers logical residuals consistent with theoretical or practical knowledge. Therefore, this paper focuses on the intermediate solution and combines it with the parsimonious solution.

**Table 5:** Results of conditional variable combination analysis.

**Types of solutions**	**Condition combinations**	**Original coverage**	**Unique coverage**	**Consistency**
Complex solution	V1^*^V2^*^V3	0.801029	0.0574614	0.805162
	V1^*^V2^*^V4	0.772727	0.0574614	0.821404
	V2^*^V3^*^V4	0.743568	0.0283019	0.763877
	~V1^*^V2^*^~V3^*^~V4	0.141509	0.0283019	0.829146
	Result coverage		0.915094	
	Result consistency		0.826839	
Intermediate solution	V1^*^V2^*^V3	0.801029	0.0574614	0.805162
	V1^*^V2^*^V4	0.772727	0.0574614	0.821404
	V2^*^V3^*^V4	0.743568	0.0283019	0.763877
	~V1^*^V2^*^~V3^*^~V4	0.141509	0.0283019	0.829146
	Result coverage		0.915094	
	Result consistency		0.826839	
Parsimonious solution	~V1	0.283019	0.0849056	0.829146
	V2^*^V3	0.829331	0.0574614	0.743274
	V2^*^V4	0.801029	0.0574614	0.736593
	Result coverage		0.971698	
	Result consistency		0.823038	

Based on the condition combination analysis of the outcome variable of the CEPCC enhancement, in terms of unique coverage, the two combinations with the highest explanatory power for the outcome variable were the infrastructure completeness ^*^ community self-organizing ability ^*^ stability of regional economic development and the infrastructure completeness ^*^ community self-organizing ability ^*^ redundancy of community resources. These combinations had a coverage of 0.0574614, indicating that they had the highest explanatory power for the enhancement of community epidemic prevention and control capability. The explanatory power for the combinations of community self-organizing ability ^*^ redundancy of community resources ^*^ stability of regional economic development and ~infrastructure completeness ^*^ community self-organizing ability ^*^~ redundancy of community resources ^*^~stability of regional economic development was the lowest, at 0.0283019. In terms of consistency, the combination of ~infrastructure completeness ^*^ community self-organizing ability ^*^~ redundancy of community resources ^*^~stability of regional economic development had the highest consistency, at 0.829146, followed by the combination of infrastructure completeness ^*^ community self-organizing ability ^*^ stability of regional economic development, with a consistency of 0.821404. The combination of community self-organizing ability ^*^ redundancy of community resources ^*^ stability of regional economic development had the lowest consistency, at 0.763877. Looking at the results of the intermediate solution, both consistency and coverage were greater than the theoretical value of 0.8, indicating that all four combinations in the intermediate solution were major contributing factors to the enhancement of the CEPCC.

By comparing the nested relationship between the parsimonious solution and the intermediate solution, the core conditions of the solution were identified. When a particular conditional variable appears in both the parsimonious solution and the complex solution, a core condition is considered. However, when a conditional variable is only present in the complex solution, a peripheral condition is considered. The final configuration analysis results are shown in Table 6, which clearly displays the relative importance of each state variable in the configuration results. In the table, the symbols “
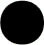
” or “•” indicate the existence of the condition, and “
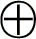
” or “⊕” indicate the absence of the condition, while a blank space implies that the presence of this condition variable is insignificant for the outcome variable. “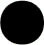 or 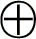
” denote a core condition, whereas “• or ⊕” refer to a peripheral condition.

**Table 6:** Configuration analysis results.

**Conditions**	**Configuration solution**			
	**Pathway 1**	**Pathway 2**	**Pathway 3**	**Pathway 4**
Infrastructure completeness	•	•		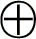
Community self-organizing ability	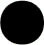	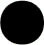	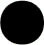	•
Redundancy of community resources	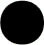		•	⊕
Stability of regional economic development		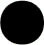	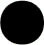	⊕
Original coverage	0.801029	0.772727	0.743568	0.141509
Unique coverage	0.0574614	0.0574614	0.0283019	0.0283019
Consistency	0.805162	0.821404	0.763877	0.829146
Total consistency	0.826839			
Total coverage	0.915094			

Table 6 summarizes the four intermediate solution configurations for affecting the CEPCC. The table presents the core and peripheral conditions for each pathway, as well as the unique coverage, original coverage and consistency information for each configuration. The unique coverage represents the proportion of cases that can be explained by each pathway combination.

As shown in Table 6, the consistency levels of the individual pathways that promote the enhancement of the CEPCC are relatively high, with pathway 3 having the lowest level of 0.763877. Meanwhile, the total consistency value for all paths is as high as 0.826839, indicating the effectiveness of the empirical analysis. Furthermore, the total coverage value is 0.915094, implying that these four configuration pathways can explain the effectiveness of the CEPCC in 92% of cases, indicating a high degree of explanatory power. Based on the four configurations presented in Table 6, which reflect the four pathways for the enhancement of the CEPCC, this study proceeds to conduct a detailed analysis and exploration of each path.

Configuration pathway 1 (V1^*^V2^*^V3): This configuration is based on the core conditions of community self-organization ability and community resource redundancy. It is suggested that the improvement of the CEPCC can be facilitated by the presence of infrastructure completeness, strong community self-organizing ability, and high redundancy of community resources. Approximately 80% of cases can be explained by this pathway, with about 5% of cases only covered by it. A typical representative case is the S5 (Building a Four-Level Joint Defense Model of “Party Organization + Comprehensive Governance Center + Grid + Double-Linked Households” to Strengthen the “Community Defense Line” in Epidemic Prevention and Control). In the case, the regional economic level of Burqin County is relatively weak compared to other highly developed regions. However, after the outbreak of the epidemic, self-organization based on communities grew rapidly, making up for the short-term failure of the government and market. Under the guidance of the government, the local community quickly established an emergency prevention and control team to accurately assess the epidemic situation in the community and take scientific and reasonable community prevention and control measures. And by integrating and coordinating social resources, personnel allocation and material reserves, the community has effectively addressed the basic living needs of their residents, including material needs, prevention and control needs, and medical needs.

Configuration pathway 2 (V1^*^V2^*^V4): This configuration is based on the core conditions of community: self-organization ability and regional economic development stability. It is suggested that the enhancement of CEPCC can be facilitated through the presence of infrastructure completeness, strong community self-organizing ability, and stability of regional economic development. Approximately 77% of cases can be explained by this pathway, with about 5% of cases only covered by it. A typical representative case is S15 (“Whole New Epidemic Prevention” Overcomes Difficulties, Creating a High-quality Answer Sheet for Lihua Community). In the face of the sudden outbreak of the epidemic, the Lihua Community quickly formed an anti-epidemic team composed of community leaders, party members, police officers, and residents. This community collaborated with social forces to conduct joint prevention and control measures, as well as comprehensive investigations to guarantee the eradication of potential dangers. The masses and social organizations were mobilized to participate in the grid-based community management. Based on the grid management of the streets, the “1+2+N” linkage was implemented. By implementing policies, the community has facilitated the resumption of production by enterprises and resolved issues related to employment and income for residents.

Configuration pathway 3 (V2^*^V3^*^V4): This configuration suggests that the enhancement of the CEPCC can be facilitated through the presence of strong community self-organizing ability, high redundancy of community resources, and stability of regional economic development. Approximately 74% of cases can be explained by this pathway, with about 2% of cases only covered by it. Typical representative case is S20 (Writing a New Chapter in the City's United Fight Against the Epidemic). In this case, the Jinputao Community committee, properties, residents, and other relevant parties collaborated to ensure the safety and wellbeing of the residents. The community made significant efforts to safeguard the daily lives and travel of residents, through the implementation of a closed management system, 24-h duty, nucleic acid testing, and procurement of the necessary materials. The community has addressed the issue of inadequate community service resources by bolstering the community service team and volunteer team. To help residents return to normal life with the improvement of the epidemic prevention and control, the community has organized and released six batches of recruitment information amounting to almost 3,000 jobs, utilizing the economic advantages of the city to assist residents in employment. With government grants, social contributions and organized community efforts, the community was able to prevent the spread of the epidemic and reduce the loss of life and property to a certain extent.

Configuration pathway 4 (~V1^*^V2^*^~V3^*^~V4): This configuration suggests that even with weak infrastructure completeness, moderate redundancy of community resources, and slow regional economic development, the enhancement of the CEPCC can still be facilitated with strong community self-organizing ability. Approximately 14% of cases can be explained by this pathway, with about 2% of cases only covered by it. A typical representative case is S18 (Establishing the “Three-Dynamic” Mechanism to Reinforce the Front-line Defense Network in Hitting the Epidemic). In the case, the Haijiao subdistrict fully mobilized party members, volunteers and masses to establish a comprehensive prevention and control network. Relying on community grid workers, the organization implemented a zoning management system for roads, communities, and grids, along with a three-level linkage mechanism. Additionally, an internal patrol team was established for the community, and measures including dynamic monitoring and daily three-level inspections were adopted for important isolated individuals in the area. A responsibility system of party members was carried out to provide daily disinfection for crucial areas such as corridors and elevators. In summary, it formed an organizational model of “party organization + property management + social organization” for community epidemic prevention and control.

#### 3.4.3 Robustness test

Based on related research, the evaluation standards were adjusted by configuring the outcome variables and the results were recalculated using fsQCA 3.0 software (41). The consistency threshold was adjusted from 0.8 to 0.82, and the calculations were repeated using fsQCA3.0 software. The results of the conditional combination analysis were essentially identical to those obtained when the consistency threshold was 0.8. While there were changes in coverage and consistency, the four influence pathways were still present, indicating the robustness and reliability of the results of this study. Therefore, it can be concluded that the pathways listed in Table 6 have a lasting effect on improving the CEPCC.

## 4 Conclusions and recommendations

### 4.1 Conclusions

This study is grounded in the theory of community resilience and employs the fsQCA research methodology to examine typical cases of COVID-19 prevention and control in 20 urban communities across China. The research findings are as follows.

Firstly, the enhancement of the CEPCC is a result of various factors. Drawing upon the theory of community resilience, four salient factors have been identified, including the infrastructure completeness, the community self-organizing ability, the redundancy of community resources, and the stability of regional economic development. Communities are urged to conduct robust analyses and assessments based on their actual situations and unique contextual factors to harness their technological, organizational, economic, and social strengths while addressing weaknesses and gaps.

Secondly, there are various combinations of pathways to enhance the CEPCC. Based on the unique contextual factors of each community, the optimal combination of conditions can be formulated to facilitate the enhancement of the CEPCC.

Thirdly, the self-organizing ability of communities is an essential condition. Regardless of community infrastructure, resource redundancy, and regional economic development levels, communities in the fight against the epidemic require the support of a strong and organized community with scientific, rational, and timely leadership. Community self-organizing capacity is a critical factor in enhancing the CEPCC.

### 4.2 Recommendations

This study identifies four pathways to improve the CEPCC and confirms the validity of the data results through case studies. Based on the research findings, we make the following recommendations to help communities strengthen their ability to respond to public health emergencies.

Firstly, it is recommended to strengthen community infrastructure construction to ensure basic public services. The impact of complete community infrastructure, as highlighted in Configuration pathway 1 and Configuration pathway 2, and community resource redundancy, as emphasized in Configuration pathway 1 and Configuration pathway 3, are crucial in enhancing the CEPCC. In terms of emergency prevention and control, it is necessary to strengthen community medical facilities by integrating community medical resources, establishing a complete diagnosis and treatment mechanism, and improving community medical services and emergency response capacities. Community information construction should be reinforced to develop a unified epidemic prevention information platform and provide timely updates on epidemic dynamics, preventive measures, and public health knowledge to enhance the residents' preventive awareness. Community patrols and personnel management should also be strengthened by establishing comprehensive health archives and registration management systems, monitoring and controlling the flow of personnel within the community, and timely identifying, reporting, and isolating individuals who may be infected with the virus. Furthermore, a community material reserve system should be established, and an emergency material management plan implemented, ensuring timely and adequate supply of epidemic prevention supplies such as masks, sanitizers, and other daily necessities. Finally, it is necessary to cultivate a volunteer team familiar with community life, with strong responsibility, and good coordination skills to provide high-quality community services and improve the CEPCC.

Secondly, it is recommended to enhance community self-organizing ability and autonomy. Community self-organizing ability is mentioned in all configuration pathways, and even when lacking support from the other three conditional variables in Configuration pathway 4, it can still be an independent condition for enhancing the CEPCC. Therefore, cultivating and strengthening community self-organizing ability is key to enhancing the CEPCC. To strengthen community self-organizing ability, above all, a community self-organizing mechanism should be established, including the development of the community leadership team, improvement of the community organizational structure, clarification of responsibilities and work processes, preparation of community epidemic prevention emergency plans, and emergency material reserves and personnel deployment. This ensures that the community can respond promptly and orderly to epidemic prevention work. Next, a scientific decision-making mechanism should be established, providing scientific decision-making support to community administrators and residents through professional institutions, expert consultations, and other means, helping them make more scientific and reasonable decisions. Residents should also be involved in decision-making processes through representative meetings and community councils, enhancing their sense of involvement and responsibility. Lastly, community mobilization and organizational abilities should also be enhanced by mobilizing community members to participate in emergency team building, and improving the community's volunteer team, enhancing the cohesiveness and combat effectiveness of the community organization. This would stimulate the residents' participation and sense of responsibility, improving the overall performance of community epidemic prevention and control.

Thirdly, it is recommended to improve the level of urban economic development to strengthen regional resource mutual assistance and coordination. The data results from Configuration pathway 2 and Configuration pathway 3 show that the speed and stability of regional economic development have a certain impact on the strength of the CEPCC. Regions with higher economic levels have relatively stronger epidemic prevention and control capacities with better material conditions, equipment and medical resources that enable them to effectively combat outbreaks. Furthermore, these regions have a relatively strong community organizing and coordinating capacity, which enables them to better organize and mobilize resources, coordinate various parties and strengthen disease prevention work. According to the case distribution, communities that have been recognized as “excellent cases of national epidemic prevention and control” are mostly located in economically developed regions such as Eastern coastal provinces or provincial capitals. The improvement of urban economic development can promote resource integration and coordinated development among regions, improving resource allocation and utilization efficiency.

## 5 Limitations and future studies

Due to the restrictive nature of the research methodology and the case information, this paper did not set up a control group for comparative analysis, and failed to compare in depth the differences in different regions, times and groups, which may weaken the accuracy and reliability of the findings to a certain extent. Therefore, we will follow up these cases in our future research, combining methods such as observational research, in-depth interviews, and setting up a comparison group, to explore more deeply and comprehensively the mechanism of action between the influencing factors and the CEPCC.

## Data availability statement

The original contributions presented in the study are included in the article/supplementary material, further inquiries can be directed to the corresponding author.

## Author contributions

RS: Conceptualization, Methodology, Project administration, Writing – original draft, Writing – review & editing. BL: Data curation, Formal analysis, Software, Writing – original draft, Writing – review & editing. YZ: Data curation, Methodology, Software, Writing – original draft, Writing – review & editing.

## References

[1] Yang Q Yang D Li P Liang S Zhang Z. A bibliometric and visual analysis of global community resilience research. Int J Environ Res Public Health. (2021) 18:10857. 10.3390/ijerph182010857 34682602 PMC8535544

[2] Zhang Q Li JK. Resilient governance: challenges and explorations faced by grassroots communities in responding to compound risks. Tribune Study. (2022) 6:76–84. 10.16133/j.cnki.xxlt.2022.06.015

[3] Meng Y Wang X Dong P Yang Y Wang K Yan X. Comparative analysis of prevention and control measures toward COVID-19 epidemic between Shanghai and Beijing. Front Publ Health. (2023) 11:1121846. 10.3389/fpubh.2023.1121846 37139394 PMC10149736

[4] Zhang X Yang S. “A community system”: a critical foundation for the epidemic prevention and control of SARS-CoV-2. Int J Health Plann Manage. (2020) 35:1246–9. 10.1002/hpm.3005 32677114 PMC7405393

[5] Zhang R Yuan Y Li H Hu X. Improving the framework for analyzing community resilience to understand rural revitalization pathways in China. J Rural Stud. (2022) 94:287–94. 10.1016/j.jrurstud.2022.06.012

[6] Cui P Zou P Ju X Liu Y Su Y. Research progress and improvement ideas of anti-epidemic resilience in China's urban communities. Int J Environ Res Public Health. (2022) 19:15293. 10.3390/ijerph192215293 36430012 PMC9690367

[7] Holling CS. Resilience and stability of ecological systems. Annu Rev Ecol Syst. (1973) 4:1–23. 10.1146/annurev.es.04.110173.000245

[8] Peacock WG Brody SD Seitz WA Merrell AV Zahran S Harriss RC. Advancing the Resilience of Coastal Localities: Implementing and Sustaining the Use of Resilience Indicators. Final Report Prepared for the Coastal Services Center and The National Oceanic and Atmospheric Administration. College Station, TX: Hazard Reduction and Recovery Center (2010).

[9] Bruneau M Chang SE Eguchi RT Lee GC O'Rourke TD Reinhorn AM. A framework to quantitatively assess and enhance the seismic resilience of communities. Earthquake Spectra. (2003) 19:733–52. 10.1193/1.1623497

[10] Coles E Buckle P. Developing community resilience as a foundation for effective disaster recovery. Austr J Emerg Manag. (2004) 19:6–15. 10.3316/informit.375435145094637

[11] Wang TY Zhao JB. The scientific outlook on development and disease prevention and control work. Chin J Publ Health. (2007) 2:254–5. 10.3321/j.issn:1001-0580.2007.02.071

[12] Dempsey K Jain S Clezy K Bradd P. Implementation of a successful infection prevention and control governance structure and capacity building strategies during COVID-19 pandemic—a brief report. Am J Infect Control. (2022) 50:1278–9. 10.1016/j.ajic.2022.08.017 35839961 PMC9273515

[13] Zhao X Jin A Hu B. How do perceived social support and community social network alleviate psychological distress during COVID-19 lockdown? The mediating role of residents' epidemic prevention capability. Front Publ Health. (2022) 10:763490. 10.3389/fpubh.2022.763490 35509511 PMC9058058

[14] Cui P Liu Y Ju X Gu T. Key influencing factors and optimization strategy of epidemic resilience in urban communities—a case study of Nanjing, China. Int J Environ Res Public Health. (2022) 19:9993. 10.3390/ijerph19169993 36011626 PMC9408670

[15] Galbusera L Cardarilli M Giannopoulos G. The ERNCIP survey on COVID-19: emergency & business continuity for fostering resilience in critical infrastructures. Saf Sci. (2021) 139:105161. 10.1016/j.ssci.2021.105161 34720423 PMC8545769

[16] Chu Z Cheng M Song M. What determines urban resilience against COVID-19: city size or governance capacity? Sustain Cit Soc. (2021) 75:103304. 10.1016/j.scs.2021.103304 34540567 PMC8437392

[17] Buliva E Elhakim M Tran Minh NN Elkholy A Mala P Abubakar A. Emerging and reemerging diseases in the World Health Organization (WHO) Eastern Mediterranean Region—progress, challenges, and WHO initiatives. Front Publ Health. (2017) 5:276. 10.3389/fpubh.2017.00276 29098145 PMC5653925

[18] Mostafavi E Ghasemian A Abdinasir A Nematollahi Mahani SA Rawaf S Salehi Vaziri M. Emerging and re-emerging infectious diseases in the WHO Eastern Mediterranean region, 2001–2018. Int J Health Pol Manag. (2022) 11:1286–300. 10.34172/ijhpm.2021.13 PMC9808364 33904695

[19] Norris FH Stevens SP Pfefferbaum B Wyche KF Pfefferbaum RL. Community resilience as a metaphor, theory, set of capacities, and strategy for disaster readiness. Am J Community Psychol. (2008) 41:127–50. 10.1007/s10464-007-9156-6 18157631

[20] Miles SB. Foundations of community disaster resilience: well-being, identity, services, and capitals. Environ Hazards. (2015) 14:103–21. 10.1080/17477891.2014.999018

[21] Mayunga JS. Understanding and applying the concept of community disaster resilience: a capital-based approach. In: A Draft Working Paper Prepared for the Summer Academy for Social Vulnerability and Resilience Building. Munich (2007). p. 1–16.

[22] Cutter SL Barnes L Berry M Burton C Evans E Tate E. A place-based model for understanding community resilience to natural disasters. Glob Environ Change. (2008) 18:598–606. 10.1016/j.gloenvcha.2008.07.013

[23] Renschler CS Frazier AE Arendt LA Cimellaro GP Reinhorn AM Bruneau M. Developing the ‘PEOPLES' resilience framework for defining and measuring disaster resilience at the community scale. In: Proceedings of the 9th US National and 10th Canadian Conference on Earthquake Engineering. Toronto, ON (2010). p. 25–9.

[24] Joerin J Shaw R Takeuchi Y Krishnamurthy R. The adoption of a climate disaster resilience index in Chennai, India. Disasters. (2014) 38:540–61. 10.1111/disa.12058 24905710

[25] Wilson GA. Community resilience, globalization, and transitional pathways of decision-making. Geoforum. (2012) 43:1218–31. 10.1016/j.geoforum.2012.03.008

[26] Alshehri SA Rezgui Y Li H. Delphi-based consensus study into a framework of community resilience to disaster. Natural Hazards. (2015) 75:2221–45. 10.1007/s11069-014-1423-x

[27] Qasim S Qasim M Shrestha RP Khan AN Tun K Ashraf M. Community resilience to flood hazards in Khyber Pukhthunkhwa province of Pakistan. Int J Disast Risk Reduct. (2016) 18:100–6. 10.1016/j.ijdrr.2016.03.009

[28] Chong NO Kamarudin KH Abd Wahid SN. Framework considerations for community resilient towards disaster in Malaysia. Proc Eng. (2018) 212:165–72. 10.1016/j.proeng.2018.01.022

[29] Almutairi A Mourshed M Ameen RFM. Coastal community resilience frameworks for disaster risk management. Natural Hazards. (2020) 101:595–630. 10.1007/s11069-020-03875-3

[30] Gao EX. Balanced governance: how can normalized epidemic prevention and control succeed? A sample of epidemic control in a mega city in China. J Publ Manag. (2022) 1:1–12. 10.16149/j.cnki.23-1523.20211228.001

[31] Gao W Zhang Y Yin G. Identifying conditions for a third dose intention of COVID-19 vaccination in college students: a fuzzy-set qualitative comparative analysis. Front Publ Health. (2022) 10:932243. 10.3389/fpubh.2022.932243 36033777 PMC9411792

[32] Mahoney J Goertz G. The possibility principle: choosing negative cases in comparative research. Am Polit Sci Rev. (2004) 98:653–69. 10.1017/S0003055404041401

[33] Schneider CQ Wagemann C. Set-Theoretic Methods for the Social Science: A Guide to Qualitative Comparative Analysis. Cambridge: Cambridge University Press (2012).

[34] Fisher RE Bassett GW Buehring WA Collins MJ Dickinson DC Eaton LK. Constructing a Resilience Index for the Enhanced Critical Infrastructure Protection Program (No. ANL/DIS-10-9). Decision and Information Sciences. Argonne, IL: Argonne National Laboratory (ANL) (2010).

[35] Mash R Goliath C Perez G. Re-organising primary health care to respond to the Coronavirus epidemic in Cape Town, South Africa. Afri J Prim Health Care Fam Med. (2020) 12:1–4. 10.4102/phcfm.v12i1.2607 33181873 PMC7669993

[36] Farsalinos K Poulas K Kouretas D Vantarakis A Leotsinidis M Kouvelas D. Improved strategies to counter the COVID-19 pandemic: lockdowns vs. primary and community healthcare. Toxicol Rep. (2021) 8:1–9. 10.1016/j.toxrep.2020.12.001 33294384 PMC7713637

[37] Zhao T Wu Z. Citizen–state collaboration in combating COVID-19 in China: experiences and lessons from the perspective of co-production. Am Rev Publ Admin. (2020) 50:777–83. 10.1177/0275074020942455

[38] Zamboni LM. Theory and metrics of community resilience: a systematic literature review based on public health guidelines. Disast Med Public Health Prep. (2017) 11:756–63. 10.1017/dmp.2017.22 29280421

[39] Zhang P Wu ZJ Hou DY. Innovation of urban community governance in China: explanation of dynamic factors and types — fuzzy set qualitative comparative analysis based on 42 experimental areas. Social Stud. (2020) 2:81–9.

[40] Rihoux B Ragin CC. Configurational Comparative Methods: Qualitative Comparative Analysis (QCA) and Related Techniques. Thousand Oaks, CA: Sage Publications (2008).

[41] Fiss PC. Building better causal theories: a fuzzy set approach to typologies in organization research. Acad Manag J. (2011) 54:393–420. 10.5465/AMJ.2011.60263120

